# Exploring the association between unintended pregnancies and unmet contraceptive needs among Ugandan women of reproductive age: an analysis of the 2016 Uganda demographic and health survey

**DOI:** 10.1186/s12884-023-06222-z

**Published:** 2024-02-08

**Authors:** Daniel Asrat, Andrew Copas, Adesina Olubukola

**Affiliations:** 1 University College of London, London, UK; 2 Institute for Life and Earth Sciences Including Health and Agriculture (PAULESI), Pan African University, Oyo, Nigeria; 3 Institute for Global Health, Faculty of Pop Health Sciences, University College of London, London, UK; 4 Department of Obstetrics & Gynaecology College of Medicine and Pan African University Institute for Life and Earth Sciences Including Health and Agriculture (PAULESI), University of Ibadan, Oyo, Nigeria

**Keywords:** Unintended/Unwanted/Unplanned pregnancy, Reproductive health, Uganda, Unmet need for contraception

## Abstract

**Background:**

Unintended pregnancy and unmet contraceptive needs pose significant public health challenges, particularly in developing nations, where they contribute to maternal health risks. While previous research has explored determinants of unintended pregnancies, there remains a gap in understanding the association between unplanned pregnancies and unmet contraceptive needs among Ugandan women of reproductive age. This study aimed to assess unmet contraceptive needs and their correlation with unintended pregnancies and other factors in Uganda, utilizing a nationally representative sample.

**Methods:**

Data was extracted from the 2016 Uganda Demographic Health Survey (UDHS), a cross-sectional survey conducted in the latter half of 2016. The study encompassed 18,506 women aged 15–49 with a history of at least one prior pregnancy. The primary outcome variable was the planning status of the most recent pregnancy, while the principal independent variable was unmet contraceptive need. Additional variables were controlled in the analysis. Data analysis was performed using STATA version 17, involving descriptive analysis, cross-tabulation, chi-square testing, and logistic regression. Statistical significance was set at *p* < 0.05.

**Results:**

A substantial proportion of women reported unintended pregnancies (44.5%), with approximately 21.09% experiencing an unmet need for contraception. In the adjusted model, women with unmet contraceptive needs had 3.97 times higher odds of unintended pregnancy (95% CI = 3.61–4.37) compared to those with met contraceptive needs. Significant factors linked to unintended pregnancies included women's age, place of residence, household wealth status, decision-making authority regarding contraceptive use, educational attainment, husband's occupation, and educational level.

**Conclusion:**

This study revealed that both the rate of unintended pregnancies and unmet contraceptive needs in Uganda exceeded the global average, warranting urgent policy attention. Addressing unmet contraceptive needs emerges as a potential strategy to curtail unintended pregnancies. Further qualitative research may be necessary to elucidate the sociocultural and behavioral determinants of unwanted pregnancies, facilitating context-specific interventions.

## Introduction

Unintended pregnancy is a pregnancy that is either unwanted (the pregnancy occurred when neither parent wanted a child) or mistimed (the pregnancy occurred at an inappropriate or unplanned time [1]. Unintended pregnancy remains a significant public health challenge, particularly in low- and middle-income countries (LMICs), due to its negative socioeconomic and health effects on mothers and children [1]. According to the World Health Organization (WHO), each year 74 million women worldwide have unintended pregnancies, leading to 25 million unsafe abortions and 47,000 maternal deaths in LMICS [2]. The issue of unintended and poorly spaced pregnancies is a major concern for women’s health and well-being in sub-Saharan Africa (SSA). According to a 2022 study, the prevalence of unintended pregnancy in SSA was 33.9%, with 11.2% being unwanted pregnancies and 22.1% being mistimed pregnancies [3].

Global and regional trends and patterns of unintended pregnancy according to the latest estimates by the Guttmacher Institute and the United Nations Population Division, the global rate of unintended pregnancy declined from 79 per 1,000 women aged 15–49 in 1990–1994 to 64 per 1,000 women in 2015–2019 [4]. However, there were significant regional and subregional variations in the trends and levels of unintended pregnancy. The highest rates of unintended pregnancy were found in Africa (101 per 1,000 women), followed by Latin America and the Caribbean (76 per 1,000 women), Asia (59 per 1,000 women), Europe (44 per 1,000 women), and North America (42 per 1,000 women) [3, 5]. Within Africa, eastern Africa had the highest rate of unintended pregnancy (116 per 1,000 women), followed by central Africa (107 per 1,000 women), western Africa (94 per 1,000 women), southern Africa (77 per 1,000 women), and northern Africa (61 per 1,000 women) [3]. The regional and subregional differences in unintended pregnancy rates reflect the variations in contraceptive prevalence and unmet need for contraception, as well as the social and cultural factors that influence women’s reproductive choices and behaviors.

Unintended pregnancy is a risk factor for negative physical and mental health outcomes for the mother, husband, and children, as well as the socioeconomic characteristics of the community [2]. Several studies have shown that unintended pregnancy is associated with unsafe abortion and obstetric complications [6], suicide, depression, and delayed initiation of prenatal care for the mother [7]. Thus, unplanned pregnancy is a determinant of maternal mortality, especially in countries where abortion is illegal [8]. Moreover, unintended pregnancy has negative effects on children, resulting in increased neonatal and post-neonatal mortality, inadequate childhood vaccinations and stunting, inadequate parental care, and lower educational attainment [9]. Additionally, the economic empowerment of women can be significantly affected, as unplanned pregnancies may limit educational and career opportunities, hindering financial independence. Family financial stability is also jeopardized, as unplanned additions to the family may strain resources and hinder the ability to meet the needs of existing members [10].

Unintended pregnancy may also be a major obstacle to achieving the SDGs 4 and 8 for education and economic growth in Africa, as 50% of pregnancies among teenagers were unintended, with half of them ending up being unsafe abortions [4]. As a result, using contraception can reduce the number of unintended pregnancies, lowering the risk of women that has unsafe abortions. Studies show that averting unplanned pregnancy might save 26 million abortions, 600,000 neonatal deaths, and 104 thousand pregnancy-related deaths globally [2, 11]. Therefore, preventing unwanted pregnancy is a central focus of many interventions aimed at lowering maternal and neonatal mortality [11].

One of the main factors that contributes to unwanted pregnancy is the unmet need for contraception, which refers to the gap between women’s reproductive intentions and their contraceptive use [5]. Women who want to delay their next pregnancy for at least a few years or stop having children but are not using a contraceptive method are considered to have an unmet need for contraception [5]. This results in women being exposed to the risk of pregnancy at an unplanned time. According to a statistic, 90% of unplanned pregnancies are caused by lack of access to contraception [12]. A study found that 214 million women had unmet family planning needs and 70% of unmet need occurred in developing countries, which resulted in 23 million women being at risk of having an unplanned pregnancy [6, 11]. However, approximately 308 million unwanted pregnancies can be prevented annually using modern contraceptives [2]. Therefore, addressing unmet need for contraception might reduce the risk of unwanted pregnancy and improve maternal and child health.

Other factors that contribute to unwanted pregnancy include women’s discontinuation of contraceptives, confusion about their fertility goals, fear of sexual assault, decision of contraceptive use, contraceptive failure, and sociocultural/economic factors [7, 12]. For example, women who discontinue contraceptives due to side effects, stock-outs, cost, or partner’s opposition may experience unintended pregnancies [12]. Women who are unsure about their fertility preferences or change their mind after becoming pregnant may also report their pregnancies as unintended [4, 12]. Women who face sexual violence or coercion may have limited control over their reproductive decisions and may not be able to use contraceptives effectively or consistently [5, 7]. Women who lack access to quality reproductive health services may not receive adequate information, counseling, or methods that suit their needs and preferences [7, 12]. Women who do not have autonomy over their contraceptive use may face barriers from their partners, families, communities, or health providers [7]. Women who use contraceptives may still experience unintended pregnancies due to method failure or user error [12, 13]. Finally, women’s sociocultural/economic factors such as age, place of residence, religion, age of first birth, perception of fertility, gender rights (sexual abuse), etc. may influence their reproductive choices and behaviors [7].

Uganda is one of the countries in Sub-Saharan Africa that faces a high burden of unintended pregnancy and its adverse consequences. According to the 2014 Uganda Demographic Health Survey (UDHS), 42% of pregnancies in Uganda were unintended, with 24% being mistimed and 18% being unwanted [8]. Unintended pregnancy poses serious challenges for reproductive health and family planning in Uganda, where maternal mortality ratio is still high at 336 per 100,000 live births, contraceptive prevalence rate is low at 39%, unmet need for family planning is high at 28%, and total fertility rate is high at 5.4 children per woman [8]. Reducing unintended pregnancy could have significant benefits for improving maternal and child health outcomes, as well as enhancing social and economic development in Uganda.

The Ugandan government established a national family planning service with the goal of reducing unmet need for family planning to 10% and increasing the user’s modern contraceptive prevalence rate to 50% by 2020 [14] because they recognize the importance of family planning in economic development and reducing unplanned pregnancy related to maternal mortality and unsafe abortion. According to a recent report, 26% of married women and 20.5% of all women in Uganda have unmet need for family planning [15]. In addition, Uganda has one of the lowest rates of contraceptive use in East Africa [16]. If all unmet need were eliminated, the cost of providing pregnancy-related medical care would be reduced by US$162 million [17]. The unmet need for family planning is a global challenge highlighted in the third Sustainable Development Goal (SDG), which aims to reduce the number of unplanned pregnancies and unsafe abortions by 2030 [18].

The Ugandan government has been working hard to achieve the Sustainable Development Goals (SDGs), which are relevant for improving maternal and child health, especially SDG 3 and SDG 5. SDG 3 aims to ensure healthy lives and promote well-being for all at all ages, by expanding family planning coverage, reducing the overall fertility rate, and reducing maternal and child mortality. The government’s target is to reduce the maternal mortality ratio to less than 70 per 100,000 live births by 2030. SDG 5 focuses on achieving gender equality and empowering women and girls, which supports Uganda’s efforts to prevent deaths of newborns and children under 5 years of age. The government’s target is to reduce neonatal mortality to 12 per 1,000 live births and under-5 mortality to 25 per 1,000 live births by 2030. Moreover, the goal of ensuring universal access to sexual and reproductive health-care services, family planning, and education aligns with Uganda’s commitment to provide comprehensive healthcare services and integrate reproductive health into national programs [19]. Uganda has made remarkable progress in reducing the number of maternal deaths that occur within health facilities. However, unwanted pregnancy remains a major challenge that contributes to high rates of unplanned births, unsafe abortions, maternal and child mortality in Uganda [13]. A study reported that unintended pregnancy accounted for 52% of all pregnancies in 2015 [1]. A recent survey reported that 46.2% of all pregnancies that occurred in Uganda in 2018 were unintended [15].

This study aims to fill the gap in the literature by using the most recent and representative data from the 2016 Uganda Demographic Health Survey (UDHS) to measure the prevalence of unmet need for contraception and unintended pregnancy among all women of reproductive age in Uganda. The study also aimed to investigate the relationship between unmet need for contraception and unintended pregnancy and other factors related to unwanted pregnancy, such as sociodemographic characteristics, sexual behavior, contraceptive use, fertility preferences, partner communication and domestic violence. The study was provided useful insights for policy makers and program managers who are working to improve reproductive health and family planning services in Uganda and reduce the burden of unintended pregnancy and its negative consequences.

## Methodology

### Source of the data and study design

This study used secondary data from the 2016 Uganda Demographic and Health Survey (UDHS), which is available at (https://dhsprogram.com). To access the data, a project summary was submitted to the DHS website. The survey adopted a cross-sectional design and was conducted during the second half of 2016. It aimed to collect data from a representative sample of all women aged 15 to 49 years across the country. Over the past three decades, the DHS program has developed and enhanced a standardized core questionnaire for use in various countries.

### Study population and sampling method

The 2016 UDHS sampled 697 EAs and 696 households per EA from fifteen regions and 112 districts of Uganda, using census data and two-stage selection. The survey aimed to provide indicators for the country, urban and rural areas, and regions. Of the 20,880 households planned, 10% did not respond. The survey interviewed 18,771 women, averaging 1,200 per region. The data were weighted to include only women who had a pregnancy in the last five years. The details of sampling method, study population and distribution have already been described somewhere else [20].

### Method of data collection

The UDHS data collection took place from June to December 2016. The UDHS questionnaires, adapted from standard DHS ones, suited Uganda’s demographics. The four-section questionnaires, translated into eight languages, included the Woman’s Questionnaire for this study. The pre-test field staff debriefed and revised the questionnaires. The UDHS study covered method, sampling, data collection, and quality assurance. Women aged 15 to 49 who had a pregnancy in the last five years and lived or stayed in the selected households were interviewed. The Woman’s Questionnaire asked various questions and selected specific variables based on prior research. The details of data collection and distribution have already been explained elsewhere [20].

### Measurement of variables

#### Outcome variable

Women who experienced at least one pregnancy within the previous five years were asked, "Was the last pregnancy wanted?" Responses included "Wanted then," "Wanted later," and "Wanted no more." Following similar studies, women's most recent pregnancies were categorized as "No" if reported as intended later (mistimed pregnancy) or not wanted at all (unwanted pregnancy), and as "Yes" otherwise.

#### Independent variables

Participants were questioned about the availability of contraception at the time of the survey. An inquiry was made about whether they had ever experienced unmet contraceptive needs in the past five years. Responses included "Using for limiting," "Had an unmet need for limiting," "Had an unmet need for spacing," "No unmet need," and "Using for spacing." Unmet need for contraception was coded "Yes" for "unmet need for spacing" and "unmet need for limiting," and "No" for other responses. To analyze the data, these categories were combined into "unmet need contraceptive" (Yes) and "met need" (No).

#### Selected sociodemographic and reproductive variables

Sociodemographic and reproductive factors were carefully selected as control variables. Since the variables are multivariable constructs, several as controlling factors were selected to adapt the study for potential confounders. These confounders include demographic, socioeconomic, and reproductive health characteristics. A literature review was conducted from various medical databases for studies on unintended pregnancy and its association unmet contraceptive need. In addition, the next independent variables were carefully chosen for analysis in this research based on the availability of data in the UDHS data collection.

The socio-demographic factors were merged variables into several categories and coded according to the following criteria for the purposes of analysis: Age of the respondent (15–19/ 20–29/30–39/40–49); type of domicile (rural/urban); employment status of the woman(yes/no); both education level of women and husband: illiteracy (no formal education), primary (1–5 formal education), secondary (6–12 formal education), higher (> 12 grade formal education); sex of household head (male/female) and occupation of the husband merged in two categorical variable: blue collar (agricultural, household domestic, construction and unskilled manual); white collar (professional/technical/managerial, teaching and business); type of household wealth status(poor/middle/rich).

The criteria used to analyse reproductive health factors were merged into a number of categorical variables and encoded as follows: Age of respondent experienced first childbirth (< 18/ >  = 18 years); respondents ideal adequate number of children(< 5 child or > 5child); ever had history of pregnancy termination(yes/no); ever used any contraceptive method (yes/no); decision made for visiting health care (respondent/ husband joint); decision on contraceptive use made (respondent alone or jointly with husband) and head of the household( male/female).

### Operational definitions

1. Unintended pregnancy refers to pregnancies that are either unplanned or unwanted [12]
2. Unmet need for contraception refers to women who want to delay their next pregnancy or cease childbearing but are not using any contraceptive method [5]

### Data extracting, checking and cleaning

First, we applied to the DHS Program website and selected the Uganda demography health survey 2016. We included a cover letter explaining our research purpose and got approval after a week. Then, we downloaded the datasets in STATA format and familiarized ourselves with the structure of the database. After that, we filtered and merged relevant variables datasets using identifiers such as cluster, household, and individual numbers. We created data subsets and extracted variables for our analysis and applied sample weights to adjust for the survey design. Prior to analysis, we meticulously checked the dataset for inconsistencies, outliers, and missing data. We performed detailed validation of variable ranges, categories, and frequencies. The data showed no issues with multicollinearity.

### Data analysis method

The diverse dataset was prudently analyzed, selecting only pertinent variables. Data were weighted using the complex samples procedure in STATA. Descriptive statistics (mean, standard deviation, frequency, and percentage distribution) were initially used for objective analysis. For Objectives 2 and 3, Pearson’s χ^2^ tests and cross-tabulations were employed to ascertain group differences in pregnancy intentions across various factors. Variables showing statistical significance (*p* < 0.05) in bivariate analysis were included in next multivariable logistic regression analysis to determine the effect of independent variables on the dependent variable, controlling for confounding biases. The analysis did not identify multicollinearity. STATA Statistics Version 17 was used for all analyses.

## Result

### Sociodemographic and reproductive health characteristics

Table 1 displays the sociodemographic characteristics of the sample group of 18,506 (weighted) women who had at least one pregnancy during the preceding five years. The mean age of the respondents was 27.6 years (SD 7.8), with most of them being in the age group of 20–29 (36.72%) and 30–39 (25.01%). Many women (76.34%) lived in rural areas, while only a minority (23.66%) resided in urban areas. Women had lower levels of education than their husbands, as 12% of them were illiterate compared to 7.7% of their husbands, and only 7.1% of them had tertiary education compared to 13.9% of their husbands. More than half of both women (58.8%) and husbands (52.8%) had completed primary education. Most of the husbands (75.4%) were engaged in manual labour for a living, while only a small proportion (14.6%) were engaged in white collar professions. The distribution of household wealth was skewed, as more than two-fifths of the women (42.5%) reported very low wealth, while only one-fifth (20%) reported very high wealth. Most women (73%) were employed at the time of the survey, while one-quarter (25%) were unemployed.

**Table 1:** Presents a descriptive analysis of the sociodemographic characteristics of the 2016 UHDS sample (*N* = 18,506)

Variables	Operational definition	Number	percentage
**Age of participant**	Participants' age at the time of the survey		
15–19		4,276	23.11
20–29		6,796	36.72
30–39		4,629	25.01
40–49		2,805	15.16
**Domicile**	Type location of domicile		
Urban		4,379	23.66
Rural		14,127	76.34
**Education**	Respondent's Education Level in Categories		
No education		2,071	11.19
Primary		10,893	58.86
Secondary		4,213	22.77
Higher		1,329	7.18
**Husband education**	Formal education at the husband level		
No Education		882	7.75
Primary		6,006	52.78
Secondary		2,909	25.56
Higher		1,582	13.90
**Husband occupation**	Employment type of the husband for income generation		
Blue collar		9,691	85.38
White collar		1,660	14.62
**Wealth Status**	Availability of wealth the family's status		
Poor		7,524	40.66
Middle		6,939	37.50
Rich		4,043	21.85
**Currently employment**	the respondent's employment status at the time of the survey		
Yes		13,668	73.86
No		4,838	26.14

### Reproductive health characteristics

Table 2 displays the reproductive health characteristics of the sample group of 18,506 (weighted) women who had at least one pregnancy during the preceding five years. Most of the women in the study (70.6%) had their first child after the age of 18, while only a minority (29.4%) had their first child before reaching 18 years old. More than half of the women (55.95%) who had fewer than five children reported that they had the ideal number of children for them, while the rest (44.05%) wanted more than five children. Most of the women (83%) had never terminated a pregnancy in their lifetime and only a small proportion (17%) had ever terminated a pregnancy at least once in their lifetime. More than half of the participants (55.66%) said that they were using any contraceptive method to prevent unwanted pregnancy and 44.34% of those women reported that they had never used any contraceptive method to avoid becoming pregnant. Only a minority of the women (30.2%) reported making decisions about contraception solely on their own, while most of the women (69.68%) stated that their husbands or jointly with their husbands made the decisions to use contraception. Most of the women (70%) said that the male head of household was responsible for making decisions about health care, while only a minority (30%) said that they were the head of their household.

**Table 2:** Presents a descriptive analysis of the reproductive characteristics of the 2016 UHDS sample (*N* = 18,506)

**Age at first birth**	Age at which the first birth occurred for a responder	**Numbers**	**Percentage**
< 18		5,456	29.48
> = 18		13,050	70.52
**Ideal number of children**	Opinions of respondents about the best number of children		
< 5		10,355	55.95
> = 5		8,151	44.05
**Ever had a terminated pregnancy**	History of abortion procedures		
Yes		3,328	17.98
No		15,178	82.02
**Use contraceptive**	ever used anything or tried to delay or prevent pregnancy?		
Yes		10,300	55.66
No		8,206	44.34
**Decision maker for using contraception**	Decision about contraceptive usage made by respondent alone or in conjunction with spouse		
Participant		1,294	30.32
Joint/husband		2,974	69.68
**Sex of household head**	Household head being a male figure		
Male		12,351	66.74
Female		6,155	33.26

Table 3 shows the prevalence of unintended pregnancy and unmet contraceptive need in Uganda based on the national representative survey. According to this study, 44.54% of the women reported that their last pregnancy was unintended, either mistimed or unwanted, while 55.46% of the women reported that their last pregnancy was wanted at the time. Most women (78.91%) had met their contraceptive need, meaning that they were using a contraceptive method for spacing or limiting births, while 21.09% had unmet contraceptive need, meaning that they wanted to space or limit births but were not using any contraceptive method.

**Table 3:** Prevalence of Unintended pregnancy and unmet need for contraception among women who had at least one pregnancy in the 5 years preceding the survey, UDHS 2016 (*N* = 18,506)

**Variables**	**Number**	**Percentage**
**Wanted last child**		
Intended	5,692	55.46
Unintended	4,571	44.54
**Unmet need of contraceptive**		
Yes	3,902	21.09
No	14,604	78.91

Table 4 presents the results of a chi-square analysis to examine the association between unintended pregnancy and various demographic and reproductive health characteristics. The rate of unintended pregnancy was 44.54% among the participants who had at least one pregnancy in the preceding five years. The analysis revealed that the following variables were significantly associated with unintended pregnancy: unmet need for contraception, age of the respondent, type of residence, decision maker for using contraception, level of education of the respondent, occupation of the husband, level of education of the husband, and current employment status of the respondent. On the other hand, the following variables were not significantly associated with unintended pregnancy: sex of the household head, history of pregnancy termination, use of any contraceptive method, and ideal number of children. The rate of unintended pregnancy was higher among respondents who had unmet need for contraception (54.64%), who were aged 15 to 19 (53.85%), who resided in rural areas (46.11%), whose husbands worked in manual labour jobs (46.29%), who were currently employed (45.77%), and who reported very low wealth status (48.04%). Moreover, respondents who reported that their husbands or jointly with their husbands made the decisions to use contraception (46.02%) and who had their first birth before the age of 18 (47.02%) were more likely to have an unintended pregnancy.

**Table 4:** Percentage reporting unintended pregnancy cross the sociodemographic and reproductive health related variables, UDHS 2016

**Variables**	**Last pregnancy intention status (*n* = 10,263)**		
	**Unintended %**	**Chi-square**	***P*-value**
**Age of participant**		116.98	< 0.001
15–19	58.28		
20–29	40.86		
30–39	44.39		
40–49	53.15		
**Domicile**		76.41	< 0.001
Urban	35.94		
Rural	46.68		
**Education level**		148.66	< 0.001
No education	37.63		
Primary	48.60		
Secondary	41.63		
Higher	27.20		
**Husband education**		113.95	< 0.001
No Education	26.77		
Primary	46.01		
Secondary	41.84		
Higher	35.26		
**Husband occupation**		30.94	< 0.001
Blue collar	43.19		
White collar	34.53		
**Wealth Status**		121.66	< 0.001
Poor	48.30		
Middle	45.25		
Rich	33.22		
**Currently employment**		15.37	< 0.001
Yes	45.50		
No |	40.71		
**Age at first birth**		95.69	< 0.001
< 18	50.67		
> = 18	40.78		
**Ideal number of children**		2.25	0.133
< 5	45.25		
> = 5	43.77		
**Ever had a terminated pregnancy**		0.41	0.518
Yes	43.91		
No	44.70		
**Use contraceptive**		1.1330	0.287
Yes	44.88		
No	43.74		
**Decision maker for using contraception**		10.78	0.001
Participant	42.28		
Joint/husband	48.45		
**Sex of household head**		0.75	0.38
Male	44.28		
Female	45.24		
**Unmet need of contraceptive**		822.15	< 0.001
Yes	65.44		
No	35.07		

Table 5 shows the results of a multivariate analysis of variables related to unwanted pregnancies in Uganda. Variables that were not significantly correlated in chi-square bivariate tests were excluded from the analysis. The remaining variables were included in two models: Model 1 with individual variables and Model 2 with grouped variables. The results indicated a significant link between women’s unmet contraceptive needs and unintended pregnancies. Women with unmet contraceptive needs had 3.50 times higher odds of unintended pregnancies than those with met contraceptive needs (95% CI = 3.21–3.80). This link was significant after adjusting for factors such as age, education of women and their husbands, residence type, husband’s occupation, age at first childbirth, wealth status, and decision maker for contraception (*P* < 0.001). Women aged 20–29 had 45% lower odds of unwanted pregnancies than those aged 15–19 (95% CI: 36.0%—52.6%). Rural women had 56% higher odds of unwanted pregnancies than urban women after adjusting for age, unmet contraceptive need, and education (*P* < 0.01). Women with no education had 1.33 times higher odds of unintended pregnancies than those with primary education, after adjusting for unmet contraceptive needs, age, and decision maker for contraception (*P* < 0.001).

**Table 5:** Multivariate logistic regression analysis between the factors associated with unwanted pregnancy in Uganda, UDHS 2016

**Variables**	**Model- 1**		**Model- 2**	
	**COR (95% CI)**	***P*-Value**	**AOR (95% CI)**	***P*-value**
**Age**		< 0.001		< 0.001
15–9	1		1	
20–29	0.55 (0.48—0.64		0.58 (0.50–0.68)	
30–39	0.64 (0.55- 0.75)		0.64 (0.54- 0.75)	
40–49	1.03 (0.85 1.25)		1.01 (0.83–1.23)	
**Domicile**		0.01		< 0.001
Urban	1		1	
Rural	1.56 (1.41–1.72)		1.44 (1.29 -1.59)	
**Education level**		< 0.001		< 0.001
Illiterate	1		1	
Primary	1.56 (1.38–1.77)		1.70 (1.4- 1.94)	
Secondary	1.11 (1.02- 1.36)		1.38 (1.18- 1.60)	
Higher	0.61 (0.50–0.76)		0.75 (0.60- 0.93)	
**Husband education**				
Illiterate	1		1	
Primary	2.33(1.94–2.79)	< 0.001	2.30 (1.90–2.78)	< 0.001
Secondary	1.96(1.62–2.38)		2.02 (1.65- 2.47)	
Higher	1.49v(1.20–1.84)		1.54 (1.23- 1.92)	
**Husband occupation**		< 0.001		< 0.001
White collar	1		1	
Blue collar	1.44 (1.26–1.64)		1.38(1.20–1.58)	
**Wealth Status**		0.02		< 0.01
Poor	1		1	
Middle	0.88 (0.81–0.96)		1.57(1.39–1.77)	
Rich	0.56 (0.47- 0.59)		0.61 (0.54–0.68)	
**Currently employment**		< 0.01		< 0.001
No	1		1	
Yes	1.21(1.10- 1.34)		1.24 (1.12–1.37)	
**Age at first birth**		< 0.001		0.001
< 18	1.49 (1.37–1.61)		1.49 (1.37–1.62)	
> = 18	1		1	
**Decision maker for using contraception**		0.04		0.001
Joint/ Husband	1		1	
Participant	1.28 (1.10–1.48)		1.28(1.10–1.48)	
**Unmet need of contraceptive**		0.001		< 0.001
Yes	3.50 (3.21- 3.8)		3.97 (3.61–4.37)	
No	1		1	

Women whose husbands had higher education levels had lower odds of unintended pregnancies (AOR = 16.24; 95% CI = 11.34 – 23.24) than those whose husbands had primary education, after controlling for age and unmet contraceptive need. Women with poor wealth status had 1.63 times higher odds of unintended pregnancies (95% CI: 1.45 – 1.83) than those with rich wealth status, after adjusting for unmet contraceptive needs and employment status. Women who had their first child before 18 had 1.49 times higher odds of unwanted pregnancies than those who had their first child after 18, after controlling for unmet contraceptive needs and current contraceptive use (*P* < 0.001). Women who decided on contraception on their own had 1.28 times lower odds of unintended pregnancies than those who decided with their husbands or by their husbands alone, after adjusting for age and unmet contraceptive need (95% CI: 1.10—1.48). Women who were employed had 1.24 times higher odds of unintended pregnancies than those who were not employed (OR 1.24; 95% CI: 1.12—1.37).

## Discussion

This study examined the relationship between unintended pregnancy and unmet contraceptive need, and other factors related to unintended pregnancy in Uganda, using the national sample of the UDHS2016. This study showed that the prevalence of unintended pregnancy was 44.54%, slightly higher than the global rate of 38% (Yaya et al.). The rate of unintended pregnancy increased by 7% from the 2014 UDHS. This may be due to reduced access to reproductive health care, especially modern contraception. Similar DHS surveys reported that the prevalence of unintended pregnancy was 40% in Congo, 43% in Malawi, 43% in Kenya, and 38.3% in Angola [7, 21–23]. In contrast, some studies found lower rates of unintended pregnancy in Ethiopia (26.6%), Kenya (24%), and Bangladesh (29.5%) [12, 21, 22]. The prevalence of unmet contraceptive need in this study was 21.09%, higher than the global average of 18% among women in 2019. This finding was similar to those reported in Ethiopia (19.1%) and Bangladesh (13.1%) [12, 21]. The rate of unmet contraceptive need in this study was slightly lower than in Angola (51.7%) [7]. The variation in prevalence rates could reflect differences in sociocultural factors, health care settings, time gaps between studies, and availability of reproductive health services across countries.

This study found a strong and significant link between unmet contraceptive need and unintended pregnancy. Women who had unmet contraceptive need had 3.50 times higher odds (95% CI = 3.21–3.8) of unintended pregnancy than women who had met contraceptive need. This link remained significant after adjusting for other factors, such as age, education, residence, occupation, wealth status, and decision maker for contraception, using multivariate logistic regression. Women who had unmet contraceptive need had 3.97 times higher odds (95% CI = 3.61–4.37) of unintended pregnancy than women who had met contraceptive need, after controlling for all other factors. This finding agrees with previous studies from Ethiopia, Angola, and Bangladesh [7, 12, 21], which also found unmet contraceptive need as a risk factor for unintended pregnancy. Addressing unmet contraceptive need could improve maternal and child health and contribute to the maternal health SDGs in developing countries like Uganda. The results support the idea that interventions that increase access and availability of contraceptives could reduce unintended pregnancy, and thus reduce unsafe abortion-related morbidity and mortality. There is a need to lower the cost of contraceptives, increase public awareness of their benefits, and empower women to make informed choices about their fertility and health. Comprehensive evidence-based interventions by all stakeholders are required to assess the scope and reasons of unmet contraceptive need in Uganda. Reproductive health interventions at schools and health institutions that aim to reduce unmet contraceptive need could be effective in reducing unintended pregnancy in Uganda. However, this study also found a significant difference between the prevalence of unmet contraceptive need (21.09%) and unintended pregnancy (44.54%). Therefore, unmet contraceptive need is not the only cause of unintended pregnancy, and other factors should be explored further.

This study found a significant link between unmet contraceptive need and unintended pregnancy, and other factors. For example, older women (30–40 years old) had lower odds of unintended pregnancy than younger women (15–19 years old), after adjusting for unmet contraceptive need. This agreed with previous studies from Malawi, Bangladesh, and Ethiopia [12, 21, 24], which showed that young women were more prone to unintended pregnancy than older women. This could be due to the barriers that young women faced in accessing reproductive health care, such as stigma, lack of information, and lack of autonomy. Moreover, young women had higher rates of sexual activity, fertility, and contraceptive failure, and lower rates of contraceptive awareness than older women [25].

This study also showed that urban women had lower odds of unintended pregnancy than rural women, after adjusting for unmet contraceptive need and other factors. This matched studies from Kenya, Ethiopia, and Angola [7, 21, 22], which reported that rural women had higher risks of unintended pregnancy than urban women. Women in rural areas may have more challenges in accessing reproductive health care, such as poverty, illiteracy, limited availability of services, and lack of decision-making power. In addition, rural women may have higher fertility preferences and lower contraceptive use than urban women.

This study further showed that women with low income and low education had higher odds of unintended pregnancy than those with high income and high education, after adjusting for unmet contraceptive need and other factors. Similar studies from Ethiopia and Uganda [1, 21]*,* found that poor and illiterate women were more exposed to unintended pregnancy than wealthy and educated women. In contrast, a study from Kenya [22] found no significant link between income or education and unintended pregnancy. It is possible that low income and low education limit women’s access to reproductive health information and services, as well as their ability to control their fertility. On the other hand, wealthy and educated women may have more knowledge and choices about family planning methods and services.

This study found a significant link between unmet contraceptive need and unintended pregnancy, and other factors. For example, women whose husbands worked in manual labour jobs and had low education levels had higher odds of unintended pregnancy than those whose husbands worked in white collar jobs and had high education levels, after adjusting for unmet contraceptive need and other factors. This agreed with earlier studies from Bangladesh and Ethiopia [12, 21], which showed that husbands who worked in manual labour jobs may have less knowledge and awareness of reproductive health services or more power over their wives’ fertility decisions.

Women who decided on contraception on their own had lower odds of unintended pregnancy than those who decided with their husbands or by their husbands alone, after adjusting for unmet contraceptive need and other factors. Similar studies from Ethiopia and Bangladesh [12, 21],found that women who decided independently to use contraception had lower odds of unintended pregnancy than women who decided jointly with their husbands or by their husbands alone. Therefore, women who decide when and where to seek reproductive health care services are a protective factor against unintended pregnancy. This shows that independence or participation in women’s health care decisions increases planning for future family planning needs and helps manage unintended pregnancy.

Women who had their first child before 18 and women who were unemployed had higher odds of unintended pregnancy than those who had their first child after 18 and those who were employed, after adjusting for unmet contraceptive need and other factors. This matched research from Ethiopia and Bangladesh [12, 21], which showed that women who had their first birth after 18 and who were employed had lower odds of unintended pregnancy than their counterparts. In contrast, a study from Kenya [22] found no significant link between employment and unintended pregnancy. This could be because women who had their first child before 18 may have higher fertility rates or lower contraceptive use than those who had their first child after 18. Women who were employed may face more difficulties in balancing work and family responsibilities or have less access to reproductive health care due to time or cost constraints.

## Conclusion

This study finds that unintended pregnancy and unmet contraceptive need are high and significant public health issues in Uganda, exceeding the global average. This could cause unsafe abortion and maternal mortality, as abortion is illegal in Uganda. This study indicates a strong link between unintended pregnancy and unmet contraceptive need, implying that meeting the contraceptive need would help reduce unintended pregnancy. Other factors related to unintended pregnancy are age, residence, age at first birth, education, husband’s education and occupation, wealth status, employment status, and decision maker for contraception. A family planning program that enhances access to reproductive health services and education is vital for reducing unintended pregnancy in the study population. All stakeholders should collaborate to improve the availability and quality of family planning services for the study population.

### Recommendation

Unintended pregnancy is a serious public health issue in Uganda, causing unsafe abortion and maternal mortality. This study indicates that unmet contraceptive need is the main factor for unintended pregnancy, along with other demographic and socioeconomic factors. To reduce unintended pregnancy and its consequences, we propose the following recommendations:

- Implement a national family planning program that offers contraceptive services to women before and after childbirth, especially in rural areas with higher unmet contraceptive need.
- Provide education and contraceptive counselling to women, especially those who are illiterate or have low education, to improve their knowledge and awareness of contraceptive methods and side effects.
- Introduce reproductive health education in secondary schools and provide affordable reproductive health services to adolescents, especially those under 20, to prevent teenage pregnancy.
- Increase male involvement in reproductive health services and decision-making, and promote gender equality and women’s empowerment, to reduce the impact of husbands’ occupation, education, and preferences on women’s fertility.

These recommendations could help achieve the SDGs on maternal and child health by reducing unintended pregnancy and its effects on women’s health and well-being. All stakeholders, including governments, NGOs, community leaders, health professionals, and family planning providers, have a role in this effort.

### Limitations and strength

This study has some limitations due to the secondary data. The cross-sectional survey cannot show causality between unmet contraceptive need and unintended pregnancy. The self-reported measures may have recall and social desirability biases. The data do not show the timing of unmet contraceptive need and unintended pregnancy. Women’s perception of unintended pregnancy may vary after giving birth. The data may not capture the current situation in Uganda, as the survey was done years ago. The study does not differentiate unplanned, unwanted, and mistimed pregnancies.

However, this study uses reliable data and a standard method that provide valid and useful evidence for interventions to prevent unintended pregnancy in Uganda. The large sample size and high representativeness are major strengths. This study can also inform future research in this area.

### Future research

This study proposes some avenues for future research on this topic. A longitudinal study could investigate the causal link between unmet contraceptive need and unintended pregnancy, and the factors that affect women’s attitudes and practices regarding contraception and pregnancy prevention. Another study could examine the early identification of unintended pregnancy, to address the outcomes of abortion or live birth.

## Data Availability

The data for this study was obtained from the DHS Program website, which holds the sole authority to distribute/share the datasets. The data can be downloaded through registration.: https://dhspr ogram.com/data/dataset/
